# The Fear of Pain Questionnaire: Factor structure, validity and reliability of the Italian translation

**DOI:** 10.1371/journal.pone.0210757

**Published:** 2019-01-25

**Authors:** Marialaura Di Tella, Ada Ghiggia, Silvia Testa, Lorys Castelli, Mauro Adenzato

**Affiliations:** Department of Psychology, University of Turin, Via Verdi, Turin, Italy; Universita degli Studi di Udine, ITALY

## Abstract

**Background:**

The Fear of Pain Questionnaire-III (FPQ-III) is a self-report instrument developed to assess fear of different stimuli usually causing pain. The present study aimed to construct an Italian version of the FPQ-III and examine its psychometric properties in a heterogeneous sample of Italian healthy individuals.

**Methods:**

The questionnaire was translated following the forward-backward method and completed by 511 Italian adults who met the inclusion criteria. Within 2 months of the first assessment, a subgroup of participants (***n*** = 164) was re-tested. The factorial structure of the FPQ-III was assessed by confirmatory factor analysis (CFA). To better comprehend the FPQ-III’s factorial structure, a CFA was also performed for each of the two reduced versions of the FPQ-III, namely the FPQ-Short Form and the FPQ-9. Divergent validity, test-retest reliability, and gender/age measurement invariance were also evaluated.

**Results:**

The results of the CFA revealed that the original three-factor model poorly fitted the data, but it became satisfactory after allowing correlated error terms. Concerning divergent validity, correlations between FPQ-III scores and pain intensity, depression, and anxiety were found to be positive but weak in magnitude (< .20). FPQ-III subscales and total scores showed good internal consistency and time reliability. Finally, scalar invariance was only partially obtained, whereas all the other types of invariance were fully respected both for gender and age.

**Conclusions:**

The current findings indicate that the Italian version of the FPQ-III provides valid and reliable scores for the assessment of fear of pain in the Italian population.

## Introduction

Fear of pain is considered as a relevant psychological factor in the development and maintenance of chronic pain and pain-related disability [1,2]. Individuals with a high fear of pain may present maladaptive responses to painful stimuli, which often involve situational avoidance [3].

According to the fear-avoidance model [4–6], when a person feels pain, two opposite reactions may take place. On the one hand, an avoidance response exacerbates fear; if pain is interpreted as threatening, pain-related fear evolves. Individuals may show avoidance behaviours and hypervigilance to bodily sensations, with resulting increased disability and psychological distress. On the other hand, confrontation as a response leads individuals to a reduction of fear over time; if the pain experience is not catastrophized, pain-related fear will not occur. This could lead the person to promptly deal with daily activities, with a faster recovery from painful injuries [6].

Given the fear of pain’s central role in pain management, specific measures of this construct are necessary in both clinical and research settings. The Fear of Pain Questionnaire-III (FPQ-III) is a self-report instrument that was developed specifically to assess fear of different stimuli usually causing pain [7]. It comprises 30 items from which can be derived a total and three subscale (i.e. Severe, Minor and Medical Pain) scores. Satisfactory test-retest reliability, internal consistency reliability, and predictive validity were reported [7–9]. However, regarding the three-factor structure originally proposed [7], subsequent independent confirmatory factor analyses (CFAs) indicated the model could be improved [10–12]. Indeed, most of the studies have obtained an adequate model fit only when using shorter or item-parcel versions of the FPQ-III [10,11,13,14].

The questionnaire is now available in English [7] and other languages [10,13–15]. However, an Italian translation has not yet been developed.

The present study aimed to construct an Italian version of the FPQ-III and examine its psychometric properties (i.e. the suggested three-factor structure, divergent validity with respect to pain intensity, depression and anxiety scores, and test-retest reliability) in a heterogeneous sample of Italian individuals. To better comprehend the FPQ-III’s factorial structure, a CFA was also performed for each of the two reduced versions of the FPQ-III, namely the FPQ-Short Form (FPQ-SF) [16] and the FPQ-9 [17]. We did not consider item-parcel versions of the FPQ-III because item parcelling could lead to distortion of the factor structure [18]. In particular, analysis at the parcel level could mask model misspecification (i.e. the presence of secondary loadings, error covariances) or items unreliability. Moreover, when the parcelled items share part of their error variances, this covariation is erroneously reformulated as common factor variance.

As a secondary aim, gender and age measurement invariance was assessed. Measurement invariance evaluates whether scales measure the same construct regardless of the group [19]. Gender and age group differences were found in previous studies, with women and young adults reporting higher scores on the FPQ compared to men and older people, respectively [7,10,12]. However, to ascertain if between-group differences really exist, each sample must have a similar understanding of the questionnaire’s items; that is, measurement invariance across gender and age groups needs to be established.

## Materials and methods

### Participants

Five hundred eighty-five participants were recruited for the present study. The inclusion criteria were as follows: over 18 years old, Italian mother tongue, a sufficient educational level (>5 years), and no presence or history of a neurological or severe psychiatric disorder. Five hundred eleven participants met the inclusion criteria and completed the questionnaires, making up the final sample enrolled in the study. Of the 511 final participants, 369 (72.2%) were women and 142 were men.

The study was approved by the University of Turin ethics committee and was conducted in accordance with the Declaration of Helsinki. All the participants gave their written informed consent to participate in the study.

### Procedure

The present data were collected by means of an online survey between August and December 2017.

First, the FPQ-III was translated into Italian following the back-translation method to ensure the semantic equivalence of the Italian and the English versions. Accordingly, the questionnaire was initially translated from English into Italian by two experts in the field with fluent English, and back translated by an English university lecturer with fluent Italian. The two English versions were finally compared and differences were identified and corrected (see S1 File for the final version of the Italian translation of the FPQ-III).

Next, a link to the survey was emailed to those who gave their agreement to take part in the study. Participants were asked to indicate sociodemographic and clinical information, and to complete two self-report measures (i.e. State-Trait Anxiety Inventory-Form Y2, and Beck Depression Inventory) in addition to the FPQ-III.

In order to examine test-retest reliability of the FPQ-III, within 2 months of the first administration a subgroup of participants (*n* = 164) was asked to complete the FPQ-III again.

### Measures

#### Sociodemographic and clinical information

All participants were asked to provide sociodemographic (i.e. gender, age, education level, marital status, occupation and origin) and clinical information (i.e. history or presence of psychiatric or neurological disorders and presence of pain). In particular, concerning clinical data, information about any chronic pain conditions the participants might suffer from was collected. In accordance with the definition of chronic pain [20,21], pain that has lasted for at least 3 months has been considered chronic. Also, to quantify the average intensity of pain experienced by each individual in the previous week, a Numeric Rating Scale, (NRS) ranging from 0 (*No pain*) to 10 (*Extreme pain*), was administered.

#### Fear of pain

The FPQ-III [7] is a 30-item self-report measure that assesses fear of pain associated with a series of potentially harmful stimuli. Each item is rated on a 5-point Likert scale ranging from 1 (*not at all*) to 5 (*extreme*), and the total score ranges from 30 to 150. Three subscale scores can be derived: Severe Pain, Minor Pain and Medical Pain. Each of these subscales consists of 10 items; example items include “Breaking your arm” for Severe Pain, “Cutting your tongue licking an envelope” for Minor Pain and “Receiving an injection in your arm” for Medical Pain. Satisfactory psychometric properties, including good internal consistency and test-retest reliability, have been reported in previous studies [11,14,15].

Two shortened versions of the FPQ-III have also been proposed. The FPQ-SF was first developed by Asmundson et al. [16]. It consists of 20 items and has four factorial distinct subscales: Severe Pain, Minor Pain, Injection Pain (e.g. “Having a blood sample drawn with a hypodermic needle”) and Dental Pain (e.g. “Having a tooth pulled”). Each item is rated on a 5-point Likert scale and the total score ranges from 20 to 100. The developers reported good internal consistency and construct validity for the FPQ-SF [16].

The other reduced version, the FPQ-9, was recently proposed by McNeil et al. [17] in order to provide researchers and clinicians with a quicker form of the questionnaire to be administered. It consists of 9 items from the original version and maintains the original three-factor structure (i.e. Sever Pain, Minor Pain and Medical/Dental Pain). Each item is rated on a 5-point Likert scale and the total score ranges from 9 to 45. The authors reported good psychometric properties for the FPQ-9, with an adequate model fit, high measures of internal consistency for the subscales and a high degree of correlation between the original version and the new one.

#### Anxiety

Anxiety was assessed using Form Y of the State-Trait Anxiety Inventory (STAI-Y) [22,23]. It is divided into two sections that can be used independently, each consisting of 20 items that are scored using a 4-point Likert-type scale: the STAI-Y1 assesses current feelings of apprehension, tension, nervousness and worry (state anxiety), and the STAI-Y2 evaluates persistent anxiety traits (trait anxiety). Each section has a total score ranging from 20 to 80, with higher scores indicating greater anxiety. In the present study, the STAI-Y2 for trait anxiety was administered.

The STAI-Y has shown good psychometric properties including adequate internal consistency (Cronbach’s α scores = 0.86–0.95), test-retest reliability (Pearson’s *r* = 0.31–0.86) and construct validity (Pearson’s *r* = 0.47–0.58 with the Beck Anxiety Inventory) [24]. In line with these results, in our sample the Cronbach’s alpha was excellent for the STAI-Y2 (α = 0.93).

#### Depression

The presence of depressive symptoms was assessed using the Beck Depression Inventory–II (BDI-II), one of the most widely used self-rating scales for assessing the severity of depression [25,26]. Each item represents a “symptom-attitude type” and is answered using a 4-point scale ranging from 0 (*no symptom*) to 3 (*most severe*). The total score is the sum of all the items and ranges from 0 (*no depressive symptoms*) to 63 (*severe depression*).

The BDI-II has shown good psychometric properties, with good internal consistency (Cronbach’s α score = 0.91), test-retest reliability (Pearson’s *r* = 0.93) and construct validity (Pearson’s *r* = 0.71 with the Hamilton Depression Rating Scale) [27]. In line with these results, in our sample the Cronbach’s alpha was excellent for the BDI-II (α = 0.91).

### Statistical analyses

Percentages, means and standard deviations of the sociodemographic and clinical variables were first computed to describe the characteristics of the sample. Pearson’s correlation was used to evaluate divergent validity with respect to depression, anxiety and pain intensity scores. Mean scores across gender and age groups were compared by means of the F-test and Eta-squared (η^2^) statistics.

#### Factorial structure

The factorial structure of the FPQ-III was assessed by CFA applied to the items’ covariance matrix. Given the lack of multivariate normality in the data (multivariate Mardias’ test of skewness and kurtosis = 1515.016, *p* < .001), the maximum likelihood with robust standard errors (MLR) was used as the method of estimation [28].

Three models with correlated latent variables were considered: the original 30-item three-factor model, the four-factor model based on 20 items proposed by Asmundson et al. [16] and the three-factor model based on 9 items developed by McNeil et al. [17]. Each model was estimated twice, once constraining all the error covariances to be zero and once allowing the estimation of the error covariance among those items that, from a content perspective and/or according to literature, can be expected to share part of their unique variance.

The model’s goodness of fit was evaluated by the Root Mean Square Error of Approximation (RMSEA), the Comparative Fit Index (CFI) and the Standardized Root Mean Square Residual (SRMR), employing the following rules of thumb to consider the solution as satisfactory: RMSEA < .08; CFI >.95 and SRMR < .08 [29–31].

#### Measurement invariance

Based on the literature [32–34], measurement equivalence across gender and age groups was evaluated by comparing the following set of hierarchical multi-group CFA models:

M1) configural invariance model (baseline model) in which items were constrained to load on the same factor across groups;
M2) metric invariance model in which loadings were constrained to be equal across groups;
M3) scalar invariance model in which both loadings and intercepts (i.e. the values of the items when the value of the corresponding latent variable is zero) were subjected to the equality constraints across groups;
M4) uniquenesses invariance model in which also the error variances were imposed to be equal;
M5) finally, structural invariance was assessed by imposing equality constraints on the factor variances and covariances.

Models M2 to M5 were compared to the previous model, in terms of changes in CFI (ΔCFI), RMSEA (ΔRMSEA) and Chi-square (Δχ^2^), employing the following cut-off values: .005 and .010 for ΔCFI and ΔRMSEA respectively [35], and 3 for Δ*χ*^2^/*df*, i.e. the ratio of Δ*χ*^2^ to degrees of freedom [36]. Due to the influence of sample size on the Chi-square statistic and considering that few simulation studies have investigated the performance of ΔCFI and ΔRMSEA, it was required that at least two of the three statistics exceeded the cut-off values to conclude that the hypothesis of invariance should be rejected.

#### Reliability and internal consistency

Internal consistency of the total scale and subscales was assessed by Cronbach’s α; values > .70 were considered as acceptable [37]. For the evaluation of test-retest reliability, the Intraclass Correlation Coefficient (ICC) was employed. As a rule of thumb, ICC values between .61 and .80 indicate moderate reliability, and those between .81 and .90 substantial reliability [38].

All the analyses were performed by using SPSS 24 and LISREL 8.72.

## Results

Sociodemographic and clinical characteristics of the Italian sample are presented in Table 1.

**Table 1:** Socio-demographic and clinical data of the Italian participants (*N* = 511).

	Mean (SD)	n (%)	Range
***Gender***			
** Women**		369 (72.2)	
**Age (years)**	35.9 (13.2)		18–82
***Education***			
** Basic education (ISCED 1/2)**		25 (4.9)	
** Secondary education (ISCED 3/4)**		188 (36.8)	
** Tertiary education (ISCED 5/6)**		298 (58.3)	
***Marital status***			
** Never-married**		254 (49.7)	
** Cohabitant**		72 (14.1)	
** Married**		157 (30.7)	
** Separated/divorced**		24 (4.7)	
** Widowed**		4 (0.8)	
***Occupation***			
** Student**		130 (25.4)	
** Employed**		327 (64.0)	
** Unemployed**		27 (5.3)	
** Retired**		16 (3.1)	
** Housewife**		11 (2.2)	
***Geographic area***			
** Northern Italy**		212 (41.5)	
** Central Italy**		212 (41.5)	
** Southern Italy**		38 (7.4)	
** Residence abroad**		49 (9.6)	
**Chronic Pain**		99 (19.4)	
**NRS**	1.1 (1.9)		0–9

NRS: Numeric Rating Scale for pain intensity.

The Italian translation of the FPQ items and their descriptive statistics are reported in S1 Table.

### Factorial structure

As shown at the top of Table 2, the original 3-factor model poorly fitted the data: none of the fit indices (RMSEA, CFI and SRMR) were in the acceptable range. Looking at the content of the items, it could be expected that some of them share part of their uniqueness: in the Medical subscale, four items (8,11,14 and 17) deal with injection/blood sample and two cover dental interventions (26 and 29); in the Severe subscale, two items (3 and 6) concern breaking limbs (arms and legs). When these nuisance covariations were modelled in terms of error covariances, the model fit became reasonably good.

**Table 2:** CFA models’ goodness of fit for the FPQ-III, FPQ-SF and FPQ-9.

Model	SBχ^2^	df	p	RMSEA	RMSEA CI	CFI	SRMR
***30-items three factors model***							
1. Without errors’ covariance	2336.55	402	< .001	0.097	0.093; 0.100	0.86	0.095
2. With 5 errors’ covariance^a^	1136.25	397	< .001	0.060	0.056; 0.065	0.95	0.075
***20-items four factors model***							
3. Without errors’ covariance	660.91	164	< .001	0.077	0.071; 0.083	0.92	0.078
4. With 2 errors’ covariance^b^	458.29	162	< .001	0.060	0.053; 0.066	0.95	0.063
***9-items three factors model***							
5. Without errors’ covariance	63.32	24	< .001	0.057	0.040; 0.074	0.97	0.048
6. With 1 errors’ covariance^c^	43.60	23	.006	0.042	0.022; 0.061	0.98	0.037

Note.

*a* = covariances between items 8–11, 8–14, 11–14, 3–6 and 26–29

*b* = covariances between items 3–6 and 26–29

*c* = covariances between items 14–17.

Moving to the four-factor model in which “injection” items form a separate factor (central part of Table 2), the fit indices exhibited acceptable values, especially when the error terms of two couples of items (items 3 and 6, and items 26 and 29) were allowed to covariate. Finally, with regard to the 9-item version, model fit was satisfactory, and adding the covariation between items 14 and 17 as suggested by the proposers of this short form produced a further improvement (bottom part of Table 2).

**Fig 1:**
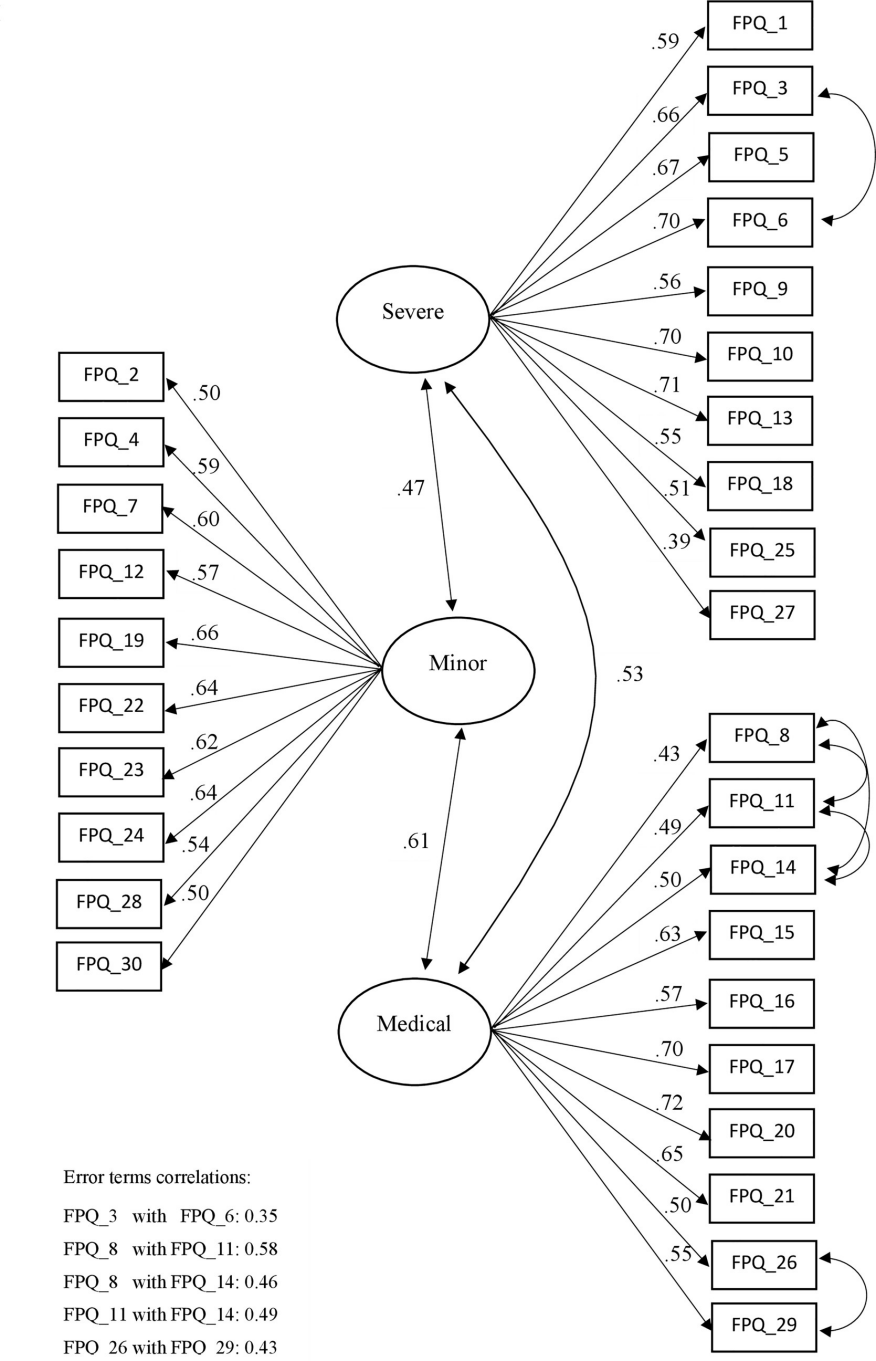
Standardized solution of the FPQ‐III three‐factor model.

From a theoretical point of view, the main difference between the 30-item three-factor model and the 20-item four-factor model concerns how the presence of a secondary factor, namely ‘Injection’, was taken into account. In the 30-item model with error covariances, the Injection factor was modelled as a nuisance factor, whereas in the 4-factor model it was conceived as a theoretical component of the general construct of fear of pain. Since the four items dealing with injection preserved substantial loadings on the Medical factor even in the presence of error covariances (see Fig 1), the original 30-item model was retained for the Italian version of the FPQ.

In the selected model (Fig 1), all the loadings were statistically significant (at *p* < .001) and of satisfactory magnitude (ranging from .39 to .72), and the correlations between the latent variables were moderate (ranging from .47 to .61). Descriptive statistics for the summed scores of the three subscales and the total scale are shown in S2 Table.

### Measurement invariance

Table 3 reports the results of the invariance tests across gender and three age groups: young adults (18 to 29 years old, *n* = 240), adults (30 to 49 years old, *n* = 168) and middle-aged/older people (aged over 49, *n* = 103). With regard to gender, configural (M1) and metric (M2) invariances were supported: Δ*χ*^2^/*df* was < 3, ΔCFI was < .005 and ΔRMSEA was < .010. Thus, the weaker form of invariance was fulfilled: items assess the same latent factors and have the same discrimination parameters across men and women. Full scalar invariance (M3) was not met: both Δ*χ*^2^/*df* and ΔCFI were out of acceptable range, signalizing that some intercept constraints had to be removed. When relaxing the equality constraint on the intercept of items 12 (*Burning your fingers with a match*) and 18 (*Being burned by a lit cigarette)*, the fit measures were adequate, and a partial scalar invariance was established. The subsequent types of invariance were also respected.

**Table 3:** Measurement and structural invariance across gender and age groups.

	Δχ^2^	df	p	Δχ^2^/df	RMSEA	CFI	ΔRMSEA	ΔCFI
***Gender***								
**Configural**					0.059	0.955		
**Metric**	49.42	27	0.005	1.830	0.059	0.953	<0.001	-0.002
**Scalar**	347.68	27	<0.001	**12.877**	0.062	0.947	0.003	**-0.007**
**Scalar**^a^	116.86	25	<0.001	**4.674**	0.061	0.950	0.001	-0.004
**Uniqueness**	65.60	35	<0.001	1.874	0.061	0.948	<0.001	-0.002
**Structural**	24.95	6	<0.001	**4.158**	0.061	0.947	<0.001	-0.001
***Age groups***								
**Configural**					0.059	0.957		
**Metric**	89.38	54	0.002	1.655	0.059	0.955	<0.001	-0.002
**Scalar**	412.05	54	<0.001	**7.631**	0.063	0.946	0.004	**-0.008**
**Scalar**^b^	159.59	48	<0.001	**3.325**	0.061	0.950	0.002	-0.004
**Uniqueness**	162.40	70	<0.001	2.320	0.062	0.945	0.001	**-0.005**
**Structural**	30.80	12	0.002	2.567	0.063	0.944	<0.001	-0.001

Note.

*a* = intercepts of FPQ18, FPQ12 free

*b* = intercepts of FPQ23 and FPQ8 freely estimated in the older group and intercepts of FPQ16, FPQ21 freely estimated in adult and older groups.

With reference to age groups, the pattern of results was similar to that obtained for gender invariance: items showed partial measurement invariance, whereas uniqueness and structural invariance was completely respected. Partial scalar invariance was obtained by removing the equality intercept constraints of items 16 (*Having an eye doctor remove a foreign particle*) and 21 (*Having a foot doctor remove a wart*) in each age group and relaxing equality constraints from items 8 (*blood sample*) and 23 (*gulping a hot drink*) in the older group only.

### Reliability and internal consistency

The FPQ-III total scale and subscales showed good internal consistency and time reliability. As shown in Table 4, Cronbach’s alphas and the intraclass correlations calculated on the subsample of 164 respondents who completed the questionnaire twice within a two-month period were all greater than .80.

**Table 4:** Internal consistency and test-retest reliability.

	Cronbach’s α	ICC	ICC 95%CI
**Severe**	.853	.828	.765-.873
**Minor**	.835	.828	.765-.874
**Medical**	.855	.917	.887-.939
**Total scale**	.906	.881	.838-.912

### Divergent validity

To get evidence of divergent validity of the instrument in the Italian context, FPQ-III subscale and total scale scores were correlated with the rating of pain intensity experienced in the previous week and the measures of depression and anxiety. As reported in Table 5, all correlations were positive but negligible in term of effect size (< .20).

**Table 5:** Pearson’s correlation with depression scores, trait anxiety scores and pain intensity scores.

FPQ	BDI	STAI-Y2	NRS
**Severe**	0.16***	0.14***	0.07
**Minor**	0.09*	0.13**	0.11*
**Medical**	0.08	0.15***	0.03
**Total scale**	0.14**	0.18***	0.08

FPQ: Fear of Pain Questionnaire; BDI: Beck Depression Inventory; STAI-Y2: State-Trait Anxiety Inventory, Form Y2; NRS: Numeric Rating Scale for pain intensity.

* p < .05

** p < .01

*** p < .001

Pain intensity was marginally correlated with the Minor Pain subscale, and BDI scores were marginally correlated with the Severe subscale and total scale scores; only STAI-Y2 scores were marginally correlated with each of the FPQ-III subscale scores and total scale score.

### Gender and age group differences

On average, women expressed a greater level of fear on the Severe and Medical Pain subscales and in terms of total score; however, the strength of this association was small, with η^2^ ranging from .03 to .05 (Table 6).

**Table 6:** FPQ mean scores across gender and age groups.

	Gender					Age groups					
	Men	Women	F	p	η^2^	Young adults	Adults	Middle-aged/ older people	F	p	η^2^
	M (SD)	M (SD)				M (SD)	M (SD)	M (SD)			
**Severe**	33.7 (7.4)	37.1 (6.5)	25.6	<0.001	0.05	36.1 (6.5)	36.3 (6.7)	35.8 (8.3)	0.2	0.841	<0.01
**Minor**	18.9 (5.8)	19.2 (5.9)	0.2	0.653	<0.01	19.1 (5.8)	18.5 (5.3)	20.1 (6.7)	2.2	0.109	0.01
**Medical**	24.3 (6.8)	27.0 (7.4)	13.7	<0.001	0.03	27.2 (7.9)	25.8 (6.9)	24.7 (6.4)	5.0	0.007	0.02
**Total scale**	76.9 (16.4)	83.2 (15.6)	16.3	<0.001	0.03	82.5 (15.9)	80.6 (15.2)	80.6 (17.9)	0.8	0.433	<0.01

With regard to age groups, only the Medical subscale showed a weak, but statistically significant means difference that, according to the Bonferroni’s post hoc analysis, occurred between the two groups of young and older respondents: older participants were less fearful about medical pain.

## Discussion

The present study aimed to construct an Italian version of the FPQ-III and examine its psychometric properties in a heterogeneous sample of Italian adults. Specifically, the suggested three-factor structure, divergent validity and test-retest reliability were assessed. In addition, gender and age measurement invariance was also evaluated.

Results indicated good to excellent levels of internal consistency for both the total scale and its subscales, as well as acceptable test-retest reliability after 2 months. Divergent validity was sustained by finding non-significant correlations with pain intensity scores (with the exception of a significant but weak correlation involving the Minor Pain subscale and pain intensity) and practically negligible associations with measures of depression and anxiety. These results, in line with previous research [11,13,14,39,40], support the specificity of the fear of pain construct, which appears to be distinct from both pain intensity and psychological distress (i.e. depression and anxiety symptoms).

Concerning the model fit, no acceptable indices were obtained when trying to replicate the original three-factor structure (Severe Pain, Minor Pain and Medical Pain). However, when a secondary factor was introduced by correlating the error term of the ‘injection’ items and a few other correlations between error terms were included, the model fit was satisfactory. These results are consistent with those obtained in previous studies [12,14]; moreover, despite their error correlation, the injection items were still good indicators of the Medical Pain subscale and the three-factor model was replicated on the 9-item short form proposed by McNeil et al. [17]. All this evidence sustains the factorial validity of the Italian version of the scale. From a theoretical point of view, the three-factor model has been preferred over the four-factor model proposed by Asmundson et al. [16]—that also fitted the data quite well—since it preserves the original constructs as the basis of the instrument.

Finally, FPQ-III’s scores showed good properties in terms of measurement invariance across gender and age groups. In both cases, the 30 items loaded in the same way on the corresponding factor (metric invariance) and had the same uniqueness (error variance); factors’ variances were also invariant across groups and this allows for interpreting the invariance of uniqueness as equality in terms of reliability of the items across groups [33]. Moreover, the relationship between factors is the same (structural invariance) despite the gender or the age group.

With regard to scalar invariance, a few items did not fulfil it. Men and women with the same latent score responded differently to the two items dealing with burning (your fingers, item 12 and your face, item 18); in particular, women scored higher on these items. For age groups, three items on the Medical Pain subscale (8, 16, 21) and one item on the Minor Pain subscale (23) were not invariant: holding the latent score constant, older people scored higher on items 21 and 23 and scored lower on items 8 and 16. These differences could be connected to the different levels of exposure to that kind of painful stimuli in the different stages of life. However, the gender and age scalar invariance can be considered tenable, being the violations were few in number.

After showing scalar measurement invariance, we compared the means of the FPQ scores for both gender and age groups. Regarding age, a statistically significant difference between the three groups (i.e. young adult, adult and middle-aged/older participants) was found only for the Medical Pain subscale, with younger individuals reporting higher scores compared to older ones. These results confirm previous findings, which showed a reduction of Medical Pain scores in middle-aged and older people [10]. In fact, it is likely that older people have been more exposed to medical situations during their life, compared to younger individuals [41].

Concerning gender, significant differences were found for the total scale and the Severe and Medical subscales scores, with women reporting higher scores than men. In line with previous studies [7,10,12], these results seem to show a more general tendency of women to respond fearfully to painful stimuli, although culture and linguistic factors may also play a role (e.g. no significant gender differences were reported for the Dutch translation of the FPQ-III [14]).

Emotions, particularly fear, has been shown to play an important role in the individual experience of both acute and chronic pain [42,43]. It is worth noting that chronic pain, specifically musculoskeletal pain, has a higher prevalence among women [44,45], who in turn appear to report more negative emotional reactions to painful situations. A broad assessment of the emotional state is therefore essential for the accurate management of pain. A self-report questionnaire, such as the FPQ-III, could represent a valid instrument to be employed in the screening of both clinical and nonclinical populations, addressing research and clinical purposes. From a scientific point of view, the use of the FPQ-III could make it possible to determine which constructs are mainly related to fear of pain, on the one hand, and to differentiate individuals with high and low fear of pain in terms of both responses to pain and subsequent outcomes, on the other. Furthermore, the possibility of having several translations of this measure can also help to understand the role that fear of pain plays in the experience of pain across different contexts and cultures, overcoming language barriers.

In a clinical setting, the instrument can provide the medical staff with useful information on how to approach each patient. Previous studies support the validity of the fear-avoidance model in the comprehension and management of chronic pain conditions [46,47]. Indeed, negative, exacerbated and ruminative thinking can bring about avoidance of those physical activities that intensify the experience of pain. These avoidant and maladaptive behaviours may in turn reinforce and/or increase psychological distress and pain-related disability [48].

Further support derives from neurofunctional studies, which suggest that a decreased connectivity between the amygdala and the periaqueductal gray, in conjunction with increased amygdala activity, might represent the neurobiological basis of how pain-related fear contributes to the exacerbation and maintenance of pain [49].

The present study has some limitations that should be considered. First, a large proportion of the sample were women. Moreover, only non-clinical participants were recruited for the study, with few individuals reporting chronic pain. Further studies with more men and with patients suffering from chronic pain should be carried out to support the generalizability of our results and establish the instrument’s predictive validity. Finally, although previous studies supported the convergent validity of the FPQ-III [7,12–14], we did not specifically evaluate this aspect in the Italian version of the questionnaire. Further studies should use additional instruments of pain-related fear or anxiety sensitivity to establish convergent validity of the questionnaire in the Italian population.

Despite these limitations, the current findings indicate that the Italian version of the FPQ-III was able to provide valid and reliable scores for the assessment of fear of pain in a heterogeneous sample of Italian individuals. The instrument could thus be applied to better understand which role the fear of pain might play in the onset and retention of chronic pain in Italian-speaking sufferers. This could allow clinicians to plan better tailored treatments specific to each individual’s needs.

## Supporting information

S1 File Fear of Pain Questionnaire–III. This is the Italian translation of the Fear of Pain Questionnaire–III.
(DOCX)

S1 Table Appendix A. Descriptive statistics for the 30 items of FPQ-III (N = 511).
(DOCX)

S2 Table Appendix B. Descriptive statistics for the summed subscales and total scores of FPQ-III (N = 511).
(DOCX)

S1 Dataset Raw data used in the current study. (XLSX)
