# Supplementary material for: The Fear of Pain Questionnaire: Factor structure, validity and reliability of the Italian translation
Source: PLoS One. 2019 Jan 25;14(1):e0210757. doi: 10.1371/journal.pone.0210757 (PMC6347221; doi:10.1371/journal.pone.0210757)
Supplement: S1 File — This is the Italian translation of the Fear of Pain Questionnaire–III. (DOCX) [file pone.0210757.s001.docx]

**FEAR OF PAIN QUESTIONNAIRE – III**

 Nome e cognome: _______________________________________ Data: __________________

ISTRUZIONI. Le affermazioni di seguito elencate descrivono esperienze dolorose. Per favore legga ogni affermazione e pensi a quanto è **spaventato** dal vivere il **dolore** associato a ciascuna affermazione. Se non ha mai sperimentato il **dolore** descritto in una delle affermazioni, risponda in base a quanta **paura** si aspetterebbe di provare se le capitasse di vivere una tale esperienza. Indichi, per ogni affermazione di seguito riportata, il numero corrispondente al livello di **paura del dolore** che proverebbe in relazione a ciascun evento descritto.

|  | | **Per nulla** | **Poco** | **Abbastanza** | **Molto** | **Moltissimo** |
| --- | --- | --- | --- | --- | --- | --- |
| **Temo il dolore associato a:** | | | | | | |
| 1. | Essere coinvolto in un incidente automobilistico | 1 | 2 | 3 | 4 | 5 |
| 2. | Mordermi la lingua mentre mangio | 1 | 2 | 3 | 4 | 5 |
| 3. | Rompermi un braccio | 1 | 2 | 3 | 4 | 5 |
| 4. | Tagliarmi la lingua leccando una busta | 1 | 2 | 3 | 4 | 5 |
| 5. | Essere colpito da un oggetto pesante sulla testa | 1 | 2 | 3 | 4 | 5 |
| 6. | Rompermi una gamba | 1 | 2 | 3 | 4 | 5 |
| 7. | Sbattere la punta del gomito | 1 | 2 | 3 | 4 | 5 |
| 8. | Fare un prelievo di sangue con un ago sottocutaneo | 1 | 2 | 3 | 4 | 5 |
| 9. | Lo sportello di una macchina che mi viene sbattuto sulla mano | 1 | 2 | 3 | 4 | 5 |
| 10. | Cadere giù per una rampa di scale in cemento | 1 | 2 | 3 | 4 | 5 |
| 11. | Ricevere un’iniezione nel braccio | 1 | 2 | 3 | 4 | 5 |
| 12. | Bruciarmi le dita con un fiammifero | 1 | 2 | 3 | 4 | 5 |
| 13. | Rompermi il collo | 1 | 2 | 3 | 4 | 5 |
| 14. | Ricevere un'iniezione nel fianco/gluteo | 1 | 2 | 3 | 4 | 5 |
| 15. | Farmi estrarre una profonda scheggia dalla pianta del piede con una pinzetta | 1 | 2 | 3 | 4 | 5 |

|  | | **Per nulla** | **Poco** | **Abbastanza** | **Molto** | **Moltissimo** |
| --- | --- | --- | --- | --- | --- | --- |
| **Temo il dolore associato a:** | | | | | | |
| 16. | Un oculista che mi rimuove un frammento incastrato nell’occhio | 1 | 2 | 3 | 4 | 5 |
| 17. | Ricevere un’iniezione in bocca | 1 | 2 | 3 | 4 | 5 |
| 18. | Essere bruciato sul viso da una sigaretta accesa | 1 | 2 | 3 | 4 | 5 |
| 19. | Tagliarmi un dito con un foglio di carta | 1 | 2 | 3 | 4 | 5 |
| 20. | Ricevere punti di sutura sul labbro | 1 | 2 | 3 | 4 | 5 |
| 21. | Un podologo che mi rimuove una verruca dal piede con uno strumento affilato | 1 | 2 | 3 | 4 | 5 |
| 22. | Tagliarmi durante la rasatura con un rasoio affilato | 1 | 2 | 3 | 4 | 5 |
| 23. | Bere una bevanda bollente prima che si sia raffreddata | 1 | 2 | 3 | 4 | 5 |
| 24. | Irritarmi entrambi gli occhi con del sapone mentre mi faccio il bagno o la doccia | 1 | 2 | 3 | 4 | 5 |
| 25. | Avere una malattia terminale che mi causa quotidianamente dolore | 1 | 2 | 3 | 4 | 5 |
| 26. | Farmi rimuovere un dente | 1 | 2 | 3 | 4 | 5 |
| 27. | Vomitare ripetutamente a causa di un’intossicazione alimentare | 1 | 2 | 3 | 4 | 5 |
| 28. | Sabbia o polvere che mi entrano negli occhi | 1 | 2 | 3 | 4 | 5 |
| 29. | Farmi trapanare un dente | 1 | 2 | 3 | 4 | 5 |
| 30. | Avere un crampo muscolare | 1 | 2 | 3 | 4 | 5 |
