# Supplementary material for: The Fear of Pain Questionnaire: Factor structure, validity and reliability of the Italian translation
Source: PLoS One. 2019 Jan 25;14(1):e0210757. doi: 10.1371/journal.pone.0210757 (PMC6347221; doi:10.1371/journal.pone.0210757)
Supplement: S1 Table — Appendix A. Descriptive statistics for the 30 items of FPQ-III (N = 511). (DOCX) [file pone.0210757.s002.docx]

**APPENDIX A – Descriptive statistics for the 30 items of FPQ-III (N=511)**

| **Item content (English/Italian)** | **M** | **SD** | **Skew.** | **Kurt.** |
| --- | --- | --- | --- | --- |
| ^a^1. Being in an automobile accident/Essere coinvolto in un incidente automobilistico | 3.7 | 1.1 | -0.5 | -0.4 |
| ^a^2. Biting your tongue while eating/Mordermi la lingua mentre mangio | 1.9 | 0.9 | 0.9 | 0.6 |
| ^a,b^ 3. Breaking your arm/Rompermi un braccio | 3.3 | 1.1 | -0.2 | -0.6 |
| ^a^4. Cutting your tongue licking an envelope/Tagliarmi la lingua leccando una busta | 2.1 | 1.1 | 0.8 | -0.1 |
| ^a^5. Having a heavy object hit you in the head/Essere colpito da un oggetto pesante sulla testa | 3.4 | 1.1 | -0.3 | -0.7 |
| ^a^6. Breaking your leg/Rompermi una gamba | 3.6 | 1.1 | -0.6 | -0.2 |
| ^a^7. Hitting a sensitive bone in your elbow-your “funny bone”/Sbattere la punta del gomito | 2.1 | 1.0 | 0.8 | 0.3 |
| ^a^8. Having a blood sample drawn with a hypodermic needle/Fare un prelievo di sangue con un ago sottocutaneo | 1.8 | 1.1 | 1.4 | 1.2 |
| ^a,b^ 9. Having someone slam a heavy car door on your hand/Lo sportello di una macchina che mi viene sbattuto sulla mano | 3.3 | 1.1 | -0.2 | -0.6 |
| ^a,b^ 10. Falling down a ﬂight of concrete stairs/Cadere giù per una rampa di scale in cemento | 3.5 | 1.1 | -0.4 | -0.5 |
| ^a^11. Receiving an injection in your arm/Ricevere un’iniezione nel braccio | 1.8 | 1.1 | 1.3 | 1.0 |
| 12. Burning your ﬁngers with a match/Bruciarmi le dita con un fiammifero | 1.9 | 0.9 | 0.8 | 0.4 |
| 13. Breaking your neck/Rompermi il collo | 4.3 | 1.0 | -1.6 | 2.3 |
| ^a,b^ 14. Receiving an injection in your hip/buttocks/Ricevere un'iniezione nel fianco/gluteo | 1.9 | 1.1 | 1.1 | 0.5 |
| 15. Having a deep splinter in the sole of your foot probed and removed with tweezers/Farmi estrarre una profonda scheggia dalla pianta del piede con una pinzetta | 2.4 | 1.1 | 0.6 | -0.2 |
| 16. Having an eye doctor remove a foreign particle stuck in your eye/Un oculista che mi rimuove un frammento incastrato nell’occhio | 3.7 | 1.2 | -0.5 | -0.6 |
| ^a,b^ 17. Receiving an injection in your mouth/Ricevere un’iniezione in bocca | 2.7 | 1.2 | 0.2 | -0.8 |
| 18. Being burned on your face by a lit cigarette/Essere bruciato sul viso da una sigaretta accesa | 3.5 | 1.1 | -0.3 | -0.5 |
| ^a,b^ 19. Getting a paper-cut on your ﬁnger/Tagliarmi un dito con un foglio di carta | 1.8 | 0.9 | 1.2 | 1.5 |
| 20. Receiving stitches in your lip/Ricevere punti di sutura sul labbro | 3.2 | 1.0 | 0.0 | -0.5 |
| ^b^ 21. Having a foot doctor remove a wart from your foot with a sharp instrument/Un podologo che mi rimuove una verruca dal piede con uno strumento affilato | 2.6 | 1.1 | 0.2 | -0.6 |
| ^a^22. Cutting yourself while shaving with a sharp razor/Tagliarmi durante la rasatura con un rasoio affilato | 1.9 | 0.9 | 1.1 | 1.1 |
| ^a,b^ 23. Gulping a hot drink before it has cooled/Bere una bevanda bollente prima che si sia raffreddata | 2.0 | 0.9 | 0.8 | 0.4 |
| ^a,b^ 24. Getting strong soap in both your eyes while bathing or showering/Irritarmi entrambi gli occhi con del sapone mentre mi faccio il bagno o la doccia | 1.5 | 0.7 | 1.5 | 2.2 |
| 25. Having a terminal illness that causes you daily pain/Avere una malattia terminale che mi causa quotidianamente dolore | 4.6 | 0.7 | -1.9 | 3.6 |
| ^a^26. Having a tooth pulled/Farmi rimuovere un dente | 3.0 | 1.2 | 0.0 | -0.8 |
| 27. Vomiting repeatedly because of food poisoning/Vomitare ripetutamente a causa di un’intossicazione alimentare | 2.9 | 1.1 | 0.2 | -0.7 |
| ^a^28. Having sand or dust blow into your eyes/Sabbia o polvere che mi entrano negli occhi | 2.0 | 0.9 | 0.8 | 0.4 |
| ^a^29. Having one of your teeth drilled/Farmi trapanare un dente | 3.0 | 1.1 | 0.1 | -0.7 |
| 30. Having a muscle cramp/Avere un crampo muscolare | 1.9 | 0.9 | 0.9 | 0.8 |

*Notes.* a) Items included in the 20-item short-form (FPQ-SF); b) items included in the 9-item short-form (FPQ-9).
