# Supplementary material for: The Fear of Pain Questionnaire: Factor structure, validity and reliability of the Italian translation
Source: PLoS One. 2019 Jan 25;14(1):e0210757. doi: 10.1371/journal.pone.0210757 (PMC6347221; doi:10.1371/journal.pone.0210757)
Supplement: S2 Table — Appendix B. Descriptive statistics for the summed subscales and total scores of FPQ-III (N = 511). (DOCX) [file pone.0210757.s003.docx]

**APPENDIX B - Descriptive statistics for the summed subscales and total scores of FPQ-III (N=511)**

|  | Min. | Max. | M | SD | Skew. | Kurt. |
| --- | --- | --- | --- | --- | --- | --- |
| Severe Pain (10 items) | 12 | 50 | 36.13 | 6.95 | -0.66 | 0.57 |
| Minor Pain (10 items) | 10 | 44 | 19.12 | 5.85 | 0.84 | 0.87 |
| Medical Pain (10 items) | 10 | 46 | 26.23 | 7.35 | 0.25 | -0.32 |
| Total Score (30 items) | 32 | 131 | 81.48 | 16.07 | 0.07 | 0.27 |
